# Resilience and its external determinants: cross-sectional survey and network analysis of parenting, trauma and stress in college students

**DOI:** 10.1192/bjo.2025.10952

**Published:** 2026-01-16

**Authors:** Hongling Zhou, Liang Zhou, Jiali Wang, Shaoling Zhong, Meng Sun

**Affiliations:** Department of Social Psychiatry, The Affiliated Brain Hospital, Guangzhou Medical University, Guangzhou, China; Key Laboratory of Neurogenetics and Channelopathies of Guangdong Province and the Ministry of Education of China, Guangzhou Medical University, Guangzhou, China; Department of Community Mental Health, The Affiliated Brain Hospital, Guangzhou Medical University, Guangzhou, China; Crown Family School of Social Work, Polity, and Practice, University of Chicago, Chicago, Illinois, USA

**Keywords:** Resilience, network analysis, emotional maltreatment, college students

## Abstract

**Background:**

Compared with well-studied internal adaptive systems, there remains a lack of comprehensive exploration of external correlated factors of resilience, as well as the way in which each ingredient of resilience is influenced.

**Aims:**

This study aims to explore the dimensional associations among resilience and several factors, including parenting rearing style, childhood trauma and negative life events.

**Method:**

A series of social demographic variables, parental rearing patterns, childhood trauma, negative life events and resilience were assessed. Multiple linear regression analysis was used to explore correlated factors of resilience, with all the above factors included in the model. Network analysis was conducted to identify the central factor and key associations, and to visualise complex interactions among resilience, parenting rearing style, childhood trauma and negative life events.

**Results:**

This cross-sectional study was conducted among 4302 freshmen (2388 females, 55.5%; mean 18.59; s.d. = 0.95) from three colleges between October and December 2020. Three key associations were discovered: ‘learning pressure and emotional control’ (*r* = −0.195, *P* < 0.05), ‘emotional neglect and family support’ (*r* = −0.129, *P* < 0.05) and ‘maternal care family support’ (*r* = 0.193, *P* < 0.05). ‘Emotional abuse’ (bridge expected influence, −0.588) was the core node of the estimated network.

**Conclusions:**

This study found that learning pressure, emotional neglect and maternal care emerged as the most critical external correlates of resilience. Emotional abuse occupies the most central position in the external correlated network of resilience. Future longitudinal research should clarify the temporal impacts of these associations, and the key factors, in the dynamic resilience system.

Resilience is defined in various ways, and has been conceptualised in three main forms: (a) a dynamic, multi-systemic process that supports individuals to regain, sustain or improve their mental health in the context of adversities;^1^ (b) an internalised trait that refers to coping and adaptation when facing stresses or adversity; and (c) positive outcomes despite serious threats to adaptation or development.^2^ These models are not distinct, but rather support and complement each other. We conceptualise resilience as a dynamic, multi-systemic process that also presents a measurable internalised trait, representing the cumulative effect of the dynamic process at a specific moment. This trait emerges as an outcome from a person’s adaptation to both external and internal contexts. Good resilience is of crucial importance to either maintaining good mental health or recovering from poor mental health conditions. This integrative framework enables us to assess relatively stable internal traits and examine their correlations with multiple adversity factors.

Adolescence and early adulthood are important periods for individuals in which to develop resilience. During this period, dramatic changes occur in both body and mind. Meanwhile, a range of mental health problems also occur at this stage, the impacts of which could persist into adulthood.^3^ Previous research has also verified the continuity of resilience with the transition to adulthood, suggesting adolescence and early adulthood to be an excellent period for development of resilience.^4^ Hence, identifying the factors associated with resilience in this population is valuable because it facilitates the determination of key factors that contribute to relatively stable resilient traits.

Several researches have focused on resilience and its influencing factors among adolescents. As a multi-systemic dynamic process, resilience is reflected by multiple processes of biological (e.g. the hypothalamic–pituitary–adrenal axis system), psychological (e.g. coping style and emotional regulation), social and ecological systems (e.g. childhood trauma and adverse life events).^5^ This implies that examination of a single factor in isolation is insufficient to fully capture the essence of resilience. A considerable body of previous research has confirmed that early life adversity, such as childhood trauma,^5^ is a key risk factor that undermines resilience. Meanwhile, recent environmental factors, such as recent positive life events,^6^ social support^7^ and family functioning,^8^ are closely associated with an individual’s current levels of resilience among adolescents and young adults. At the individual level, internal factors – including self-efficacy,^9^ positive emotion^10^ and hopefulness – have been recognised as protective factors of resilience. Among Chinese undergraduates, research shows that positive cognition, coping style and family function^11^ are important correlates of resilience. Although previous studies have provided valuable knowledge on resilience, most have focused on single-dimensional correlated factors. This hinders a comprehensive interpretation of the correlates of resilience, and goes against the consensus that resilience is a multi-system process affected by both external and internal factors. In addition, previous studies mainly conducted traditional regression analysis, which makes it difficult to reveal the interactive relationships among multiple variables. Thus, evidence is still needed to clarify how each ingredient of resilience associates with external resources from a multi-dimensional perspective.

To address the above issues, we aimed to expand current knowledge by comprehensively exploring the associations among resilience and several dimensional factors (including parenting rearing styles, childhood trauma and negative life events) in a large sample of college freshmen who fall within the age range of early adulthood. This population is not only in the key period for developing resilience, but also at high risk of a series of mental problems due to their challenging new environment,^12^ which makes them an ideal population in which to explore resilience. In addition to multiple linear regression, we also adopted network analysis – an emerging data-driven approach – in this study, which can be used to examine and visualise complex interactions in an estimated network model.^13^ While traditional regression models focus on isolated correlated factors, network analysis demonstrates superior capabilities in capturing the complex, interconnected relationships among systemic correlations, through identification of the most central code and the strongest associations among clusters of variables.^14^ This technique provides the potential for comprehensive study of the associations of resilience and its influencing factors, as well as the various relationships among these correlates and multiple ingredients of resilience.

## Method

### Procedure

This cross-sectional study was conducted in three colleges (a public university, a private university and a vocational college) in Guangzhou, Guangdong Province between October and December 2020. All freshmen enrolled in 2020 were invited to participate in the online survey. With the help of college counsellors, the self-report questionnaire was distributed to all students through Quick Response code. Considering the normal age of college entrance in China and the age criteria of adolescence and early adulthood, we set the age range of inclusion at 16–25 years.^15^ To ensure the reliability of this survey, those with a response time of <5 min were excluded. We excluded the questionnaires of postgraduate students and duplicate responses based on each student’s unique student identification (maintaining only the first response). Electronic informed consent and/or parental permission were obtained from all participants. The investigation was carried out in accordance with the Helsinki Declaration as revised in 1989, and was approved by the Ethics Committees of the Affiliated Brain Hospital of Guangzhou Medical University (2019No.037).

### Measures

#### Social demographic questionnaire

Social demographic characteristics included the following: age, gender, ethnicity, place of residence (urban, town or rural), chronic physical conditions (having at least one of the following: arthritis, angina, asthma, diabetes, visual impairment or hearing problems), personal history of mental disorders and family income. In regard to the history of mental disorders, participants were initially asked whether they had a personal history of mental disorders and were then required to choose the specific diagnosis from six options, including depressive disorder, anxiety disorder, obsessive–compulsive disorder, bipolar disorder, schizophrenia and ‘others’; if they chose the option ‘others, they would be asked to specify their diagnosis.

#### Resilience Scale for Chinese Adolescents

The Resilience Scale for Chinese Adolescents (RSCA) was developed as a psychological resilience measurement specifically for Chinese adolescents. It emphasises the examination of the ‘process’ experienced by individuals when confronted with adversity and critical stress, by exploring the process variables involved in Chinese adolescents’ stress-coping, encompassing cognitive, emotional, behavioural and support system factors. In this study, we tend to interpret the scores of resilience as measurable indicators of the relatively stable internalised characteristics of a dynamic system at a single moment, which represents the cumulative effect of the dynamic process presented at that specific time. This 27-item scale evaluates current resilience from 5 dimensions (goal focus, emotion control, positive perception, family support and interpersonal assistance). Each item is rated on a 5-point Likert scale, ranging from 0 (no match at all) to 5 (perfect match). A higher total score reflects a higher level of resilience. Although this scale was initially developed for middle school students, subsequent research has confirmed that it has good reliability and validity among Chinese college students.^16^ Cronbach’s *α* for RSCA in this study was 0.794.

#### Parental Bonding Instrument

Parenting rearing style was assessed with the Parental Bonding Instrument (PBI) in this study. PBI is designed to assess the characteristics of parenting behaviours in relation to children up to 16 years of age. There are two versions of this scale, a mother (maternal) version (PBI-M) and a father (paternal) version (PBI-F), both of which comprise 23 items and are divided into 3 factors (care, encouragement and control). Each item is scored on a 4-point Likert scale, ranging from 0 (no match at all) to 3 (perfect match). PBI has shown satisfactory validity and reliability among Chinese undergraduates.^17^ Cronbach’s *α* for PBI-M and PBI-F in the current study was 0.649 and 0.673, respectively.

#### Childhood Trauma Questionnaire

We evaluated childhood trauma up to 16 years of age with the Childhood Trauma Questionnaire (CTQ) in this study. CTQ is a self-report scale of 28 items covering emotional neglect, physical neglect, sexual abuse, emotional abuse and physical abuse. Each item is evaluated on a 5-point Likert scale, ranging from 1 (never) to 5 (very often), with higher scores indicating that traumatic events had occurred more frequently. The Chinese version of CTQ has shown acceptable reliability among college students^18^ in China. In the present study, Cronbach’s *α* for CTQ was 0.749.

#### Adolescent Self-Rating Life Event Checklist

In this study, the Adolescent Self-Rating Life Event Checklist (ASLEC) was used to assess negative life events over the past year. The scale comprises 27 negative life events that may have had a psychological effect on adolescents and is divided into 5 dimensions, including relationship pressure, learning pressure, being punished, loss and adaptation problems. Each item is scored from 1 (not at all) to 5 (extremely severe), with higher total scores representing a greater impact of negative life events. The Chinese version of ASLEC has been confirmed to have good validity and reliability among college students.^19^ Cronbach’s *α* for ASLEC in this study was 0.922.

### Statistical analysis

Statistical analyses were completed using SPSS software (version 25.0, IBM Corporation, Armonk, NY, USA; https://www.ibm.com/products/spss-statistics) and R (version 4.2.1, R Foundation for Statistical Computing, Vienna, Austria; https://www.r-project.org/) on Windows. A two-sided *P*-value <0.05 was considered statistically significant in this study. Because an online questionnaire was adopted in this study, there were no missing data.

First, descriptive statistics were performed among sociodemographic variables as well as resilience, parenting rearing style, childhood trauma and negative life events. Multiple linear regression analysis was then used to explore correlates of resilience (total scores), including all sociodemographic variables, parental rearing patterns (subscale scores of both maternal and paternal versions), childhood trauma (total scores) and negative life events (total scores) in the model. The variance inflation factor (VIF) was assessed for collinearity diagnostics before multiple linear regression analysis was performed, because multicollinearity may lead to an unreliable regression model; multicollinearity is considered as being present when VIF is >10.^20^ Finally, a network analysis was conducted to determine the strongest correlation of each component of resilience (goal focus, emotion control, positive perception, family support and interpersonal assistance), because this enables visualisation and quantification of complex associations among different variable groups.^21^

#### Network structure

We estimated the network structure of resilience, parental rearing patterns, childhood trauma and negative life events using the EBICglasso model in the R package bootnet, and network structure was visualised by the R package qgraph.^22^ Resilience, parental rearing patterns, childhood trauma and negative life events were seen as four distinct communities, with each comprising multiple ingredients assessed by the subscales (see Supplementary Table 1 available at https://doi.org/10.1192/bjo.2025.10952). These components were coded as R1–5 (resilience), P1–3 and M1–3 (maternal and parental rearing patterns, respectively), C1–5 (childhood trauma) and A1–5 (negative life events), input to the model and are represented by a node in the network structure. The nodes and edges of the network, respectively, represent the variables and the partial correlation between them.^22^ We focused on those nodes having the strongest partial correlation with those in the resilience community, and thereby recognised the strongest associations among the resilience community and other communities.

#### Bridge expected influence and key nodes

Using the bridge function in the R package networktools, we identified key nodes in the network that acted as bridges among resilience, parental rearing patterns, childhood trauma and negative life events through bridge expected influence (bridge EI). Bridge EI is the sum of the values of all edges that exist between a node and all nodes that are not in the same community. Given that the network contained positive and negative edges, bridge EI was reported as the most convincing centrality index,^23^ which is of key importance for a network’s structure and functioning.^24^

#### Network stability and accuracy

We used the R package bootnet to assess the accuracy and stability of the network. First, we assessed edge weight accuracy using a non-parametric bootstrapped procedure (1000 iterations), where larger 95% confidence intervals of edge weight suggest lower accuracy.^25^ We then used the case-dropping bootstrap procedure (1000 iterations) and the correlation stability coefficient (CS-C) to assess the stability of the bridge centrality index (bridge EI). Previous studies have suggested that CS-C should be no less than 0.25, and preferably above 0.50.^25^

## Results

### Descriptive characteristics of participants

A total of 4761 college students participated in the study, 459 of whom were excluded from the sample, including 299 who were postgraduate students, 2 with a response time <5 min, 75 who were >25 years old and 83 who had duplicate submissions (data from first submission were retained). The response rate of this study was 90.4%. All 4302 college students included were aged 16−25 years (mean 18.59 years, s.d. = 0.95). The sociodemographic characteristics of all included participants are shown in Table 1; the mean scores and standard deviations for PBI, CTQ, ASLEC and RSCA are shown in Supplementary Table 1.

**Table 1:** Sociodemographic characteristics of freshmen in the three colleges (*N* = 4302)

Variables	Category	*n* (%)
Gender	Male	1914 (44.5)
	Female	2388 (55.5)
Ethnicity	Han^a^	4148 (96.4)
	Other ethnic groups	154 (3.6)
Place of residence	Urban	1789 (41.6)
	Town	1292 (30.0)
	Rural	1221 (28.4)
Chronic physical illness^b^	Yes	448 (10.4)
Personal history of mental disorders	Yes	94 (2.2)
Family income (RMB per month)	<3000	695 (16.2)
	3000–5000	998 (23.2)
	5000–7000	722 (16.8)
	7000–10 000	771 (17.9)
	>10 000	1116 (25.9)

a. Han is the major ethnic group in China.

b. Chronic physical conditions: at least one of arthritis, angina, asthma, diabetes, visual impairment or hearing problems.

### Multiple linear regression analysis of resilience

The results of multiple linear regression are shown in Table 2. Multicollinearity was not found among the assessed variables, with VIF ranging from 1.009 to 2.512. Participants from families with higher levels of income (unstandardised regression coefficient (*B*) = 0.504, *P* < 0.001), and those with greater parental care (maternal: *B* = 0.432, *P* < 0.001; paternal: *B* = 0.463, *P* < 0.001) and greater parental encouragement (maternal: *B* = 0.413, *P* < 0.001; paternal: *B* = 0.173, *P* = 0.012), were more likely to have a higher level of resilience. By contrast, those with worse resilience were more likely to be male (*B* = −0.914, *P* = 0.007), of Han ethnicity (*B* = −3.993, *P* < 0.001) and students with a history of mental disorders (*B* = −2.871, *P* = 0.013). Stricter parental control (maternal: *B* = −0.303, *P* = 0.001; paternal: *B* = −0.278, *P* = 0.003), more negative life events (*B* = −0.182；*P* < 0.001) and more childhood trauma (*B* = −0.182, *P* < 0.001) were also associated with worse resilience.

**Table 2:** Results of multiple regression models for associated factors of resilience

Demographics	*B*	s.e.	*β*	*t*	*P*-value	95% CI
Age	−0.059	0.184	−0.004	−0.321	0.748	(−0.419, 0.301)
Gender (reference, female)	−0.914	0.339	−0.032	−2.698	0.007	(−1.579, −0.250)
Ethnicity (reference, non-Han)^a^	−3.993	0.893	−0.053	−4.470	<0.001	(−5.745, −2.242)
Place of residence (reference, rural)	−0.628	0.390	−0.020	−1.610	0.107	(−1.393, 0.137)
Chronic physical illness (reference, no)	−0.819	0.547	−0.018	−1.498	0.134	(−1.891, 0.253)
Personal history of mental disorders (reference, no)	−2.871	1.160	−0.030	−2.474	0.013	(−5.146, −0.596)
Family income	0.504	0.124	0.052	4.063	<0.001	(0.261, 0.747)
Care, mother	0.432	0.051	0.158	8.450	<0.001	(0.332, 0.532)
Care, father	0.463	0.045	0.181	10.207	<0.001	(0.374, 0.552)
Encouragement, mother	0.413	0.073	0.106	5.695	<0.001	(0.271, 0.555)
Encouragement, father	0.173	0.069	0.045	2.509	0.012	(0.038, 0.308)
Control, mother	−0.303	0.089	−0.054	−3.413	0.001	(−0.478, −0.129)
Control, father	−0.278	0.094	−0.047	−2.974	0.003	(−0.462, −0.095)
CTQ	−0.180	0.023	−0.122	−7.814	<0.001	(−0.225, −0.135)
ASLEC	−0.182	0.015	−0.160	−12.198	<0.001	(−0.211,−0.153)

*B*, unstandardised regression coefficient; *β*, standardised regression coefficient; CTQ, childhood trauma questionnaire; ASLEC, adolescent self-rating life event checklist.

a. Non-Han, other ethnic group in China.

### Network analysis

#### Network structure

**Fig. 1:**
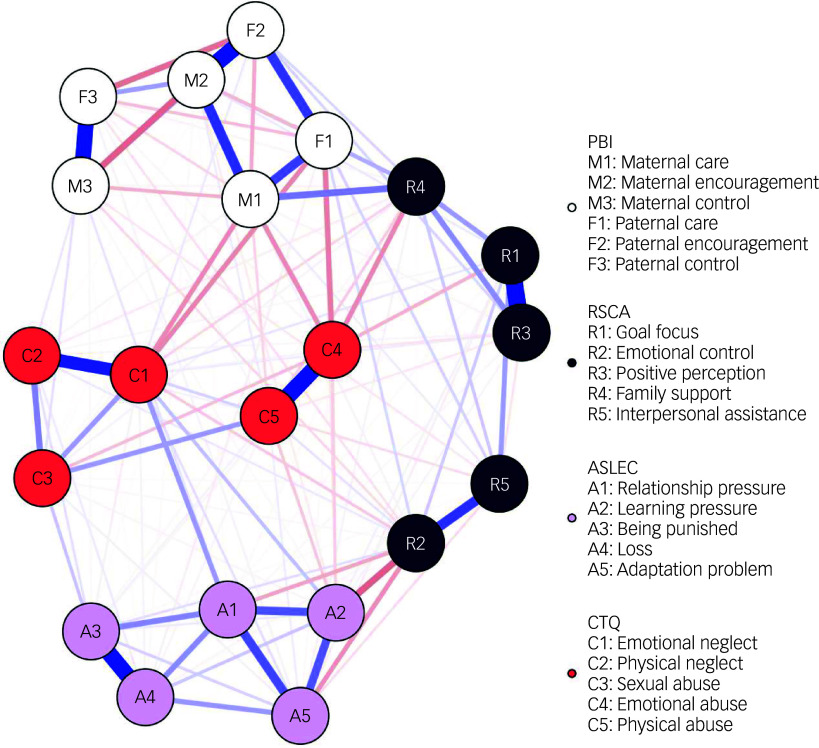
Network structure of RSCA, PBI, CTQ and ASLEC among 4302 students. Blue lines indicate positive associations, red lines indicate negative associations; the thickness of an edge represents association strength. RSCA, Resilience Scale for Chinese Adolescents; PBI, Parental Bonding Instrument; CTQ, Childhood Trauma Questionnaire; ASLEC, Adolescent Self-Rating Life Event Checklist.

The network structure of RSCA, PBI, CTQ and ASLEC is presented in Fig. 1. In the current network, 143 of 210 possible edges were set to non-zero, among which 56 were negative associations and 87 positive. Three pairs of nodes exhibited the strongest correlation between the resilience community and the communities of parental rearing patterns, childhood trauma and negative life events. Between the communities of resilience and negative life events, the strongest association found was between learning pressure (A2) and emotional control (R2) (*r* = −0.195, *P* < 0.05). For the community of resilience and childhood trauma, emotional neglect (C1) and family support (R4) shared the strongest association (*r* = −0.129, *P* < 0.05). In regard to the communities of resilience and parental rearing style, maternal care (M1) and family support (R4) showed the strongest association (*r* = 0.193, *P* < 0.05). Table 3 shows all coefficients of partial correlations among the components of RSCA, PBI, CTQ and ASLEC.

**Table 3:** Coefficients of part correlation among items of Resilience Scale for Chinese Adolescents, Parental Bond Instrument, Childhood Trauma Questionnaire and Adolescent Self-Rating Life Event Checklist

Components of the four communities	R1	R2	R3	R4	R5
A1	0	−0.097	0	−0.008	0
A2	0.021	−0.195	0.017	0	−0.010
A3	0.001	0.044	0	−0.012	0.006
A4	0.006	0.005	0.011	0	−0.014
A5	−0.024	−0.124	0	0.020	−0.041
C1	−0.083	0	−0.035	−0.129	0
C2	0	0.040	−0.028	−0.057	−0.042
C3	0	0.011	0	0.020	0.006
C4	0.037	−0.058	0.027	−0.049	0
C5	0.012	0.031	0.016	−0.027	0
F1	0	0.056	0	0.105	0.060
F2	0	0	0.057	0.052	−0.008
F3	0	−0.038	−0.001	−0.017	0
M1	0	0.021	−0.018	0.193	0.028
M2	0.031	0	0	0.068	0
M3	0	−0.044	0	−0.014	−0.031

R1, goal focus; R2, emotional control; R3, positive perception; R4, family support; R5, interpersonal assistance; A1, relationship pressure; A2, learning pressure; A3, being punished; A4, loss; A5, adaptation problem; C1, emotional neglect; C2, physical neglect; C3, sexual abuse; C4, emotional abuse; C5, physical abuse; F1, paternal care; F2, paternal encouragement; F3, paternal control; M1, maternal care; M2, maternal encouragement; M3, maternal control.

#### Bridge expected influence and key nodes

**Fig. 2:**
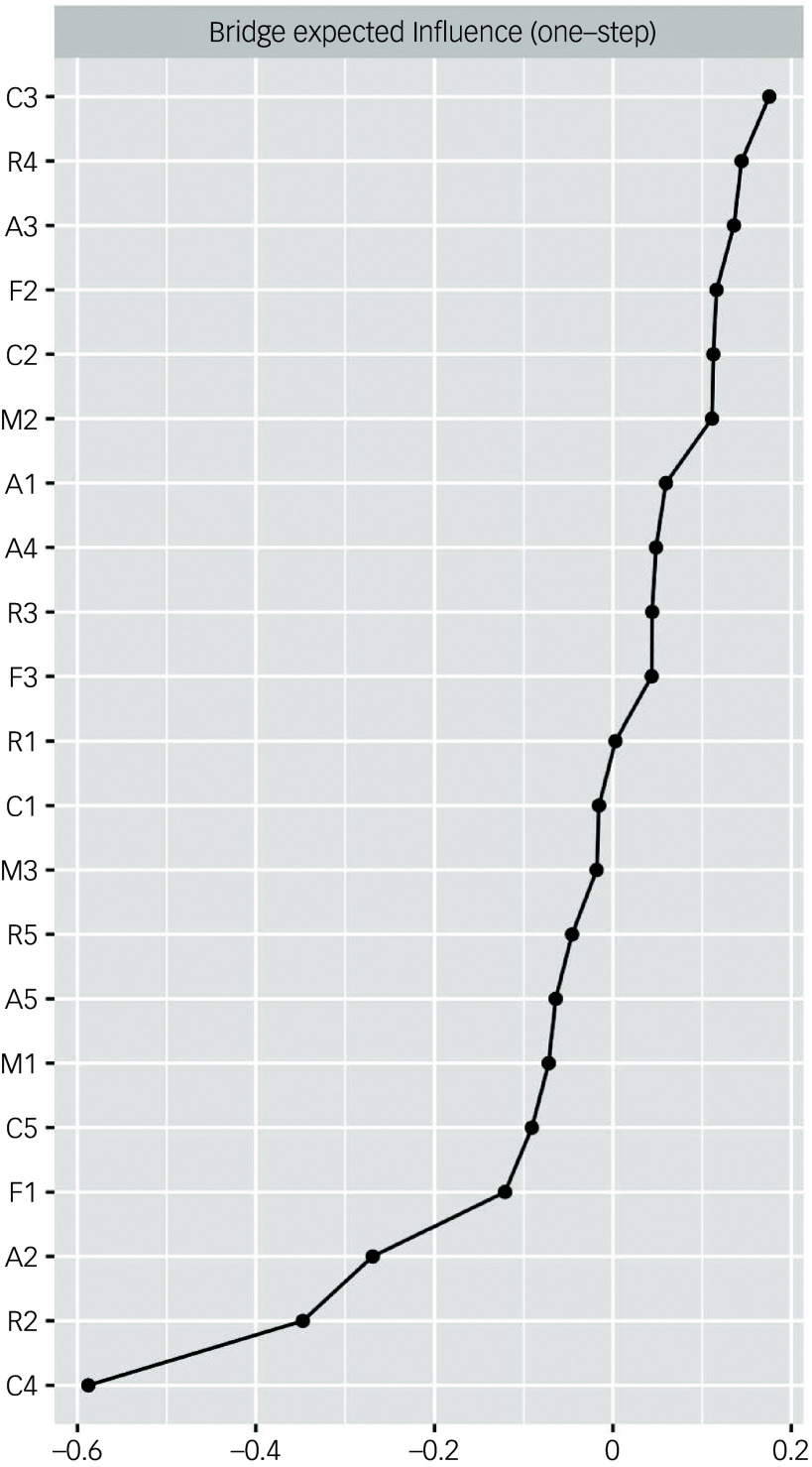
Bridge expected influence of Resilience Scale for Chinese Adolescents, Parental Bonding Instrument, Childhood Trauma Questionnaire and Adolescent Self-Rating Life Event Checklist among 4302 students. M1, maternal care; M2, maternal encouragement; M3, maternal control; F1, paternal care; F2, paternal encouragement; F3, paternal control; R1, goal focus; R2, emotional control; R3, positive perception; R4, family support; R5, interpersonal assistance; A1, relationship pressure; A2, learning pressure; A3, being punished; A4, loss; A5, adaptation problem; C1, emotional neglect; C2, physical neglect; C3, sexual abuse; C4, emotional abuse; C5, physical abuse.

Bridge EI showed that emotional abuse (bridge EI, −0.588) was the key node among all components of RSCA, PBI, CTQ and ASLEC, followed by emotional control (bridge EI, −0.347) and learning pressure (bridge EI, −0.269). The bridge expected influence of the estimated network is shown in Fig. 2.

#### Network accuracy and stability

The 95% confidence intervals of edge weights showed acceptable width, indicating that the current network structure was robust (Supplementary Fig. 1). The case-dropping bootstrap procedure suggested that the values of bridge EI and bridge strength remained stable even after elimination of different proportions of the sample (Supplementary Fig. 2). The CS-C for bridge EI was 0.75, indicating that 75% of the sample could be eliminated while maintaining a high correlation (*r* = 0.7) with the original centrality indices for the whole sample.

## Discussion

The current study comprehensively analysed the correlations among resilience, parental rearing patterns, childhood trauma and negative life events in a large sample of college freshmen, using the network analysis method. We identified three main associations within the resilience correlation network: learning pressure−emotional control, emotional neglect−family support and maternal care−family support. Furthermore, emotional abuse was identified as the key node among the network structure of RSCA, PBI, CTQ and ASLEC. Our research suggests that learning pressure, emotional neglect and maternal care are the most critical external factors related to the resilience system.

The results of multiple linear regression demonstrated that a higher number of negative life events was significantly associated with lower resilience, while network analysis further revealed that emotional control was strongly and negatively linked to learning pressure between the RSCA and ASLEC communities. Although previous research has found a close relationship between learning pressure and potential mental and physical health problems,^26^ little is known about its adverse impact on resilience. In the current study, learning pressure comprised ‘unsatisfactory academic performance’, ‘excessive academic burden’, ‘family financial difficulties’, ‘failure of expected selection’ (e.g. Merit student) and ‘enrolment pressure’. It contains both acute pressure from a sudden stressor (e.g. failure of expected selection) and long-term chronic pressure (e.g. excessive academic burden). The learning pressure−emotional control association can be explained by a previous report showing that excessive pressure can lead to emotional vulnerability^27^ and hinder the development of resilience, along with the accumulation of pressure.^28^ Cumulative and persistent perceived academic pressure leaves no room for resilience to flourish. Alternatively, low resilience can render everyday academic demands unbearably distressing. Further work is required to explore the bidirectional interplay between learning pressure and resilience in the university context.

The negatively strong link between emotional neglect and family support is in line with a previous study demonstrating that childhood emotional neglect has a significant long-term predictive effect on early-adulthood resilience.^5^ Emotional neglect means the absence of fulfilment of adolescents’ emotional needs, especially the loss of emotional support from their families. In that study, emotional neglect was assessed by several items, including ‘feeling valued’, ‘feeling loved’, ‘feeling close to each other’, ‘families caring about each other’ and ‘feeling supported and gaining strength from family’, all of which were scored in reverse. Another study concluded that there is a strong association between emotional neglect and adverse mental health outcomes.^29^ Emotional neglect in childhood has been shown to be associated with avoidant adult attachment,^30^ depression and anxiety.^31^ Neglected children suffer more from internalised symptoms than their peers exposed to other forms of abuse.^32^ Previous research also suggested that emotional neglect is linked to lower perceived social support.^30^ Our findings further verified the greatest association of emotional neglect on the family support trait of resilience among all five components of childhood trauma. In the current study, a relationship was found between emotional neglect and lack of family support, which accounts for a key part of social support. In addition, lack of family support further leads to a failure to facilitate good emotional responses and to cultivate emotional coping skills. While emotional neglect can be more invisible than physical abuse, it calls for more attention considering its strong link with resilience, especially in populations who are prone to suffering from emotional neglect from their families, such as individuals with left-behind experience.

Although parental care and parental encouragement showed correlation levels similar to those of resilience in multiple linear regression analysis, we further discovered that maternal care occupies a prominent position in the relevant resilience network. In this study, maternal care comprised 11 items (e.g. ‘understanding my problems and concerns’, ‘often smiling at me’ and ‘speaking to me in a gentle and friendly tone’), which reflect a mother being gentle, understanding and supportive of her children. Many studies have reported a positive correlation between a caring parental style and children’s mental health development.^33^ In our results, compared with parenting styles that encourage independence and freedom and interfere with and restrict children, parental care − especially maternal care − more closely connected to the trait of family support in the resilience system. These results can be understood while considering that mothers tend to take more responsibility for their children’s upbringing while fathers tend to bear more economic pressure in traditional Chinese family patterns.^34^ Additionally, previous research has also demonstrated that maternal bonding helps shape more stable resilience characteristics and styles, while impacts of paternal bonding are more variable.^35^

In the current study, emotional abuse was identified as the strongest core node among the estimated network, and the CTQ subscale was most strongly connected to the subscales of RSCA, PBI and ASLEC, demonstrating the key role of emotional maltreatment in the entire network structure. Broad associations between emotional abuse and adverse mental health outcomes have been observed in previous research,^36^ but few connections were reported on resilience. These clinical outcomes may be related to insecure attachment, and to delay or damage to psychological development and cognitive process following emotional abuse.^37^ Besides, these experiences of childhood emotional abuse strengthen the negative development of self-attitudes and inferential styles with more intrusion of negative memories, which might reflect negative inferences when dealing with stressful life events.^38^ Our results verify the findings of existing researches and further indicate the vital role of emotional abuse in the complex resilience network.^39^

Additionally, in our findings, age and place of residence were not significantly correlated with resilience. This may be attributed to the homogeneity of our sample, which consisted entirely of university freshmen. Furthermore, we found that chronic physical illness did not share significant correlation with resilience while personal history of mental disorders did. This observation supports our primary finding, that factors associated with emotional fluctuations occupy a more central role within the resilience system, while physical illness and abuse are insufficiently associated.

### Limitations

Several limitations should be noted in this study. First, its cross-sectional nature means that causality cannot be inferred. The resilience measured at single time points tends to evaluate the relatively stable internalised traits of resilience, which can appear as the cumulative effect at the specific time point but cannot capture its temporal changes as a dynamic process. Future longitudinal studies are still needed to investigate the temporal relationships among resilience, parental rearing patterns, childhood trauma and negative life events. Second, because retrospectively self-reported measures were used, recall bias cannot be eliminated and social desirability might have led to underestimation of the prevalence of childhood trauma and negative events. Diverse report forms are expected in future research. Third, because the current study was conducted among college freshmen, most of whom were from families with high socioeconomic status and had received high-level education, the findings may not adequately represent some of the most vulnerable resilience traits found in other populations. This limitation restricts the generalisability of our findings to broader adolescent or young adult populations, especially to those individuals who have not received higher education. Fourth, the resilience scale was originally developed for adolescents, which might have limited the measure for unique resilience characteristics of college students. However, we believe that the scale remains appropriate for this study because the transition from adolescence to emerging adulthood forms a continuous spectrum, and the core components of resilience remain critically important across these stages. Finally, the three main associations of the resilience network remained of modest magnitude, suggesting that potential confounding factors remain. However, this study expands current knowledge by demonstrating the central associations in the estimated network, which acknowledges the key factors with the potential to propagate wide-ranging, amplified benefits across the entire resilience system.

In conclusion, this study found that learning pressure, emotional neglect and maternal care emerged as the most critical external correlates of resilience. Emotional abuse occupies the most central position in the external correlated network of resilience. Future longitudinal research should clarify the temporal impacts of these associations and key factors in the dynamic resilience system.

## Supporting information

Zhou et al. supplementary material 1 Zhou et al. supplementary material

Zhou et al. supplementary material 2 Zhou et al. supplementary material

Zhou et al. supplementary material 3 Zhou et al. supplementary material

Zhou et al. supplementary material 4 Zhou et al. supplementary material

## Data Availability

The data that support the findings of this study are available on request from the corresponding author.

## Supplementary material

The supplementary material is available online at https://doi.org/10.1192/bjo.2025.10952
