# Supplementary figures and images for: Resilience and its external determinants: cross-sectional survey and network analysis of parenting, trauma and stress in college students
Source: BJPsych Open. 2026 Jan 16;12(1):e41. doi: 10.1192/bjo.2025.10952 (PMC12835704; doi:10.1192/bjo.2025.10952)

● Bootstrap mean    ● Sample

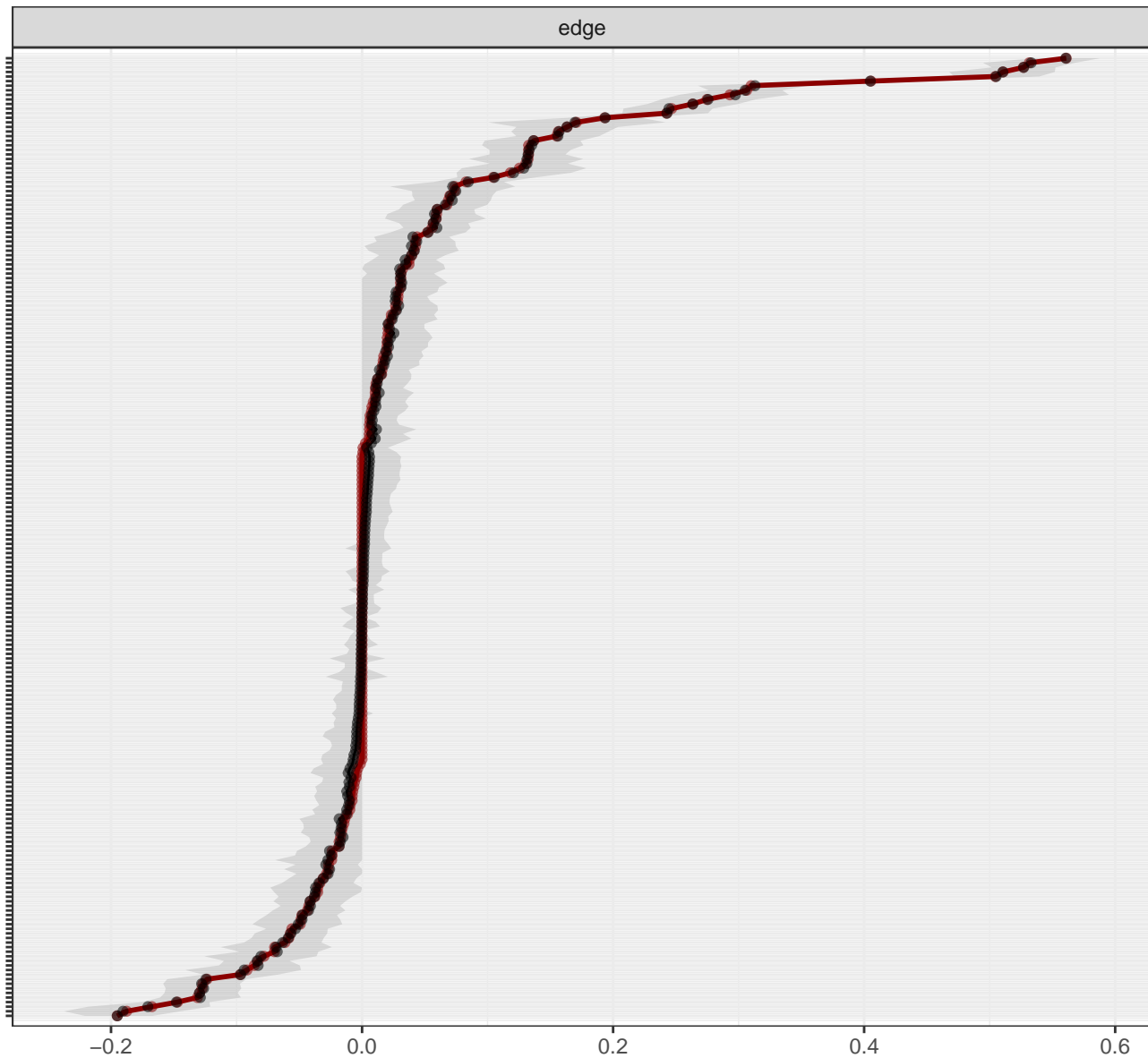

Supplement: Zhou et al. supplementary material 1 — Zhou et al. supplementary material [file S2056472425109526sup001.pdf]

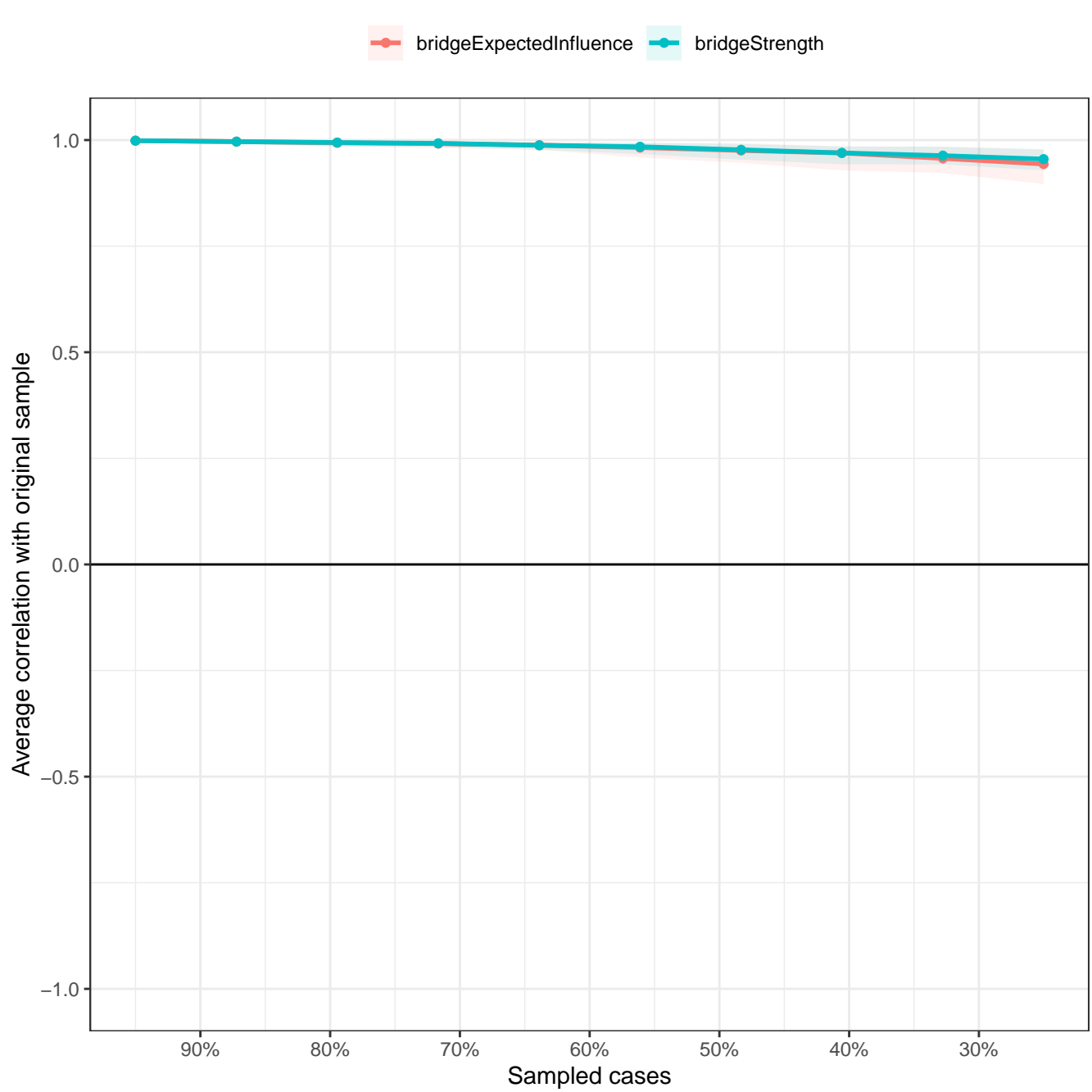

Supplement: Zhou et al. supplementary material 2 — Zhou et al. supplementary material [file S2056472425109526sup002.pdf]
