# Supplementary material for: Resilience and its external determinants: cross-sectional survey and network analysis of parenting, trauma and stress in college students
Source: BJPsych Open. 2026 Jan 16;12(1):e41. doi: 10.1192/bjo.2025.10952 (PMC12835704; doi:10.1192/bjo.2025.10952)
Supplement: Zhou et al. supplementary material 3 — Zhou et al. supplementary material [file S2056472425109526sup003.docx]

**Appendices Figure Titles and Notes**

**Supplementary Figure 1** Accuracy of the edge-weights for the current network model. **Notes:** The gray area represents the 95% Confidence Intervals of edge weights, estimated with the non-parametric bootstrap procedure. Wide intervals indicate lower stability and narrow intervals indicate higher stability.

**Supplementary Figure 2** Stability of bridge centrality indices as assessed using the case-dropping bootstrap method.
